# Stress and adolescent hippocampal neurogenesis: diet and exercise as cognitive modulators

**DOI:** 10.1038/tp.2017.48

**Published:** 2017-04-04

**Authors:** C M Hueston, J F Cryan, Y M Nolan

**Affiliations:** 1 Department of Anatomy and Neuroscience, University College Cork, Cork, Ireland; 2 APC Microbiome Institute, University College Cork, Cork, Ireland

## Abstract

Adolescence is a critical period for brain maturation. Deciphering how disturbances to the central nervous system at this time affect structure, function and behavioural outputs is important to better understand any long-lasting effects. Hippocampal neurogenesis occurs during development and continues throughout life. In adulthood, integration of these new cells into the hippocampus is important for emotional behaviour, cognitive function and neural plasticity. During the adolescent period, maturation of the hippocampus and heightened levels of hippocampal neurogenesis are observed, making alterations to neurogenesis at this time particularly consequential. As stress negatively affects hippocampal neurogenesis, and adolescence is a particularly stressful time of life, it is important to investigate the impact of stressor exposure at this time on hippocampal neurogenesis and cognitive function. Adolescence may represent not only a time for which stress can have long-lasting effects, but is also a critical period during which interventions, such as exercise and diet, could ameliorate stress-induced changes to hippocampal function. In addition, intervention at this time may also promote life-long behavioural changes that would aid in fostering increased hippocampal neurogenesis and cognitive function. This review addresses both the acute and long-term stress-induced alterations to hippocampal neurogenesis and cognition during the adolescent period, as well as changes to the stress response and pubertal hormones at this time which may result in differential effects than are observed in adulthood. We hypothesise that adolescence may represent an optimal time for healthy lifestyle changes to have a positive and long-lasting impact on hippocampal neurogenesis, and to protect against stress-induced deficits. We conclude that future research into the mechanisms underlying the susceptibility of the adolescent hippocampus to stress, exercise and diet and the consequent effect on cognition may provide insight into why adolescence may be a vital period for correct conditioning of future hippocampal function.

## Introduction

Adolescence represents a time of transition to independence during which significant lifestyle changes occur,^1, 2^ and it is believed to be a critical period for the programming of future adult behaviours.^3^ Although there are no definite markers for the adolescent period, in mice and rats adolescence is generally considered to be from post-natal day (PND) 21–60, and in humans from ages 12 to 18.^4^ Puberty, the maturation of the hypothalamic–pituitary–gonadal (HPG) axis occurs during the early adolescent period. In rodents, puberty typically occurs between PND28–42 in females and PND42–49 in males and is characterised by vaginal opening and preputial separation, respectively.^5^ In humans, puberty spans from ages 10 to 16 in girls and 11 to 17 in boys, and is characterised by the development of secondary sexual characteristics, and the onset of menses in girls^5, 6^ (please see Holder *et al.*^5^ for a pictorial representation of the relative adolescent period in rodents and humans). Significant changes in neuroendocrine, neurodevelopmental and behavioural systems occur during adolescence.^4, 7, 8, 9, 10^ For instance, there are changes in reward circuitry that render both rodent and human adolescents less sensitive to the aversive effects of drugs of abuse.^11^ Behaviourally, both rodents^7, 12, 13^ and humans^7, 14, 15, 16^ show increased risk-taking,^16^ social activity^14, 17^ and impulsivity^12, 15^ across the adolescent period. Cognitive changes have also been demonstrated at this time of the lifespan,^18^ especially in relation to executive function^19^ and cognitive control.^20^ This corresponds to neurodevelopmental changes in terms of maturation of the circuits related to learning and memory, including the hippocampus,^21, 22^ amygdala^9^ and prefrontal cortex.^18, 22^

The hippocampus is particularly altered during adolescent development, as an increased number of granule cells and overall increased volume of the hippocampal layers has been demonstrated in rodents during the adolescent period.^23, 24^ Notably, levels of hippocampal neurogenesis (the production, differentiation and integration of new neurons within the subgranular zone of the granular cell layer of the dentate gyrus (DG)),^25^ is up to four times higher during adolescence than during adulthood in rodents.^21, 26, 27, 28, 29, 30^ While the exact purpose of these newly born hippocampal cells is still controversial,^31, 32^ several lines of evidence from rodent studies have suggested that adult hippocampal neurogenesis is mandatory for stress susceptibility^33^ and optimal cognitive function,^34, 35^ at least during adulthood. Neurogenesis has been linked to cognitive behaviours that are hippocampal-dependent, and while some discrepancies are evident, overall it has been shown that tasks that require spatial memory, contextual memory or pattern separation are predominantly reliant on hippocampal neurogenesis.^36, 37^ More research is needed, however, to decipher the relative contribution of neurogenesis during adolescence to cognitive function, and especially to determine whether the types of behavioural tests that are reliant on hippocampal neurogenesis differ during this time period. While most studies that have examined the role of hippocampal neurogenesis in cognitive function have focused on the adult, it is important to consider that across the lifespan there may be differential effects. Likewise, most rodent studies examining the role of hippocampal neurogenesis in cognitive function have employed male rats; however, puberty may lead to the onset of sex differences in hippocampal neurogenesis.^38, 39^ Thus, there is still much to be explored in regards to possible sex differences in neurogenesis-associated behaviours, especially during the adolescent period.

Only a limited number of studies have examined the effects of blunted neurogenesis during adolescence on cognitive performance in later life. The results to date indicate that adolescent male rats and mice exposed to radiation, which ablates hippocampus neurogenesis, at PND21 or PND50 show a reduction in both the proliferation and survival of new neurons within the DG in adulthood,^40, 41^ as well as lasting deficits in performance in cognitive tasks such as the Morris water maze^40^ and fear-conditioning.^41^ This corresponds to findings in humans, which show that brain radiation for cancer treatment in children and adolescents produces long-term decreases in IQ scores along with other cognitive and behavioural changes.^42^ Thus, these data suggest that any changes to hippocampal neurogenesis during adolescence may have long-lasting effects on memory and learning. Altered hippocampal neurogenesis has also been linked to disorders that often emerge during adolescence or early adulthood, including depression,^43, 44, 45, 46^ schizophrenia,^46, 47, 48^ drug abuse^49, 50^ and impulse control disorders such as attention-deficit hyperactivity disorder,^51, 52^ all of which have significant cognitive as well as emotional symptoms. As such, alterations in neurogenesis during adolescence may be important in initiating the onset or development of disease.^53^

It is also important to consider other lifestyle and physiological changes that occur during the adolescent period, and how these could influence both neurogenesis and cognition. The transitional period of adolescence is a particularly stressful one,^4, 54^ with a majority of human adolescents reporting mood disturbances and anxiety.^55^ Stress has been shown to have a predominantly negative impact on hippocampal neurogenesis.^56, 57^ In particular, chronic exposure to stressful situations including psychosocial or physical stressors has been demonstrated to be detrimental to adult neurogenesis in the DG in several species including mice, rats, marmoset monkeys and tree shrews, in that it decreases cell proliferation, neuronal differentiation and cell survival.^58, 59, 60^ Related to this, chronic stress has been shown to impair hippocampal-dependent behaviours including learning and memory in rodents.^61^ As such, the stress associated with the adolescent period may produce alterations in hippocampal neurogenesis and cognitive function. Other extrinsic signals, such as physical exercise and diet, have also been identified as regulators of adult hippocampal neurogenesis in rodents.^62^ As adolescence is a vulnerable period for lifestyle influences, positive alterations in diet and exercise at this time could help to attenuate the negative impact of stressor exposure on neurogenesis and cognition.^63, 64, 65, 66, 67^

**Figure 1:**
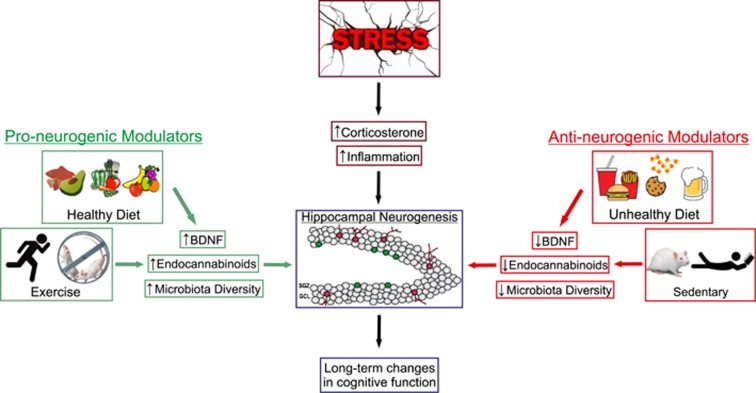
A schematic illustration of the impact of both negative and positive lifestyle factors on stress-induced alterations to neurogenesis in the dentate gyrus of the adolescent hippocampus. BDNF, brain-derived neurotrophic factor; GCL, granular cell layer; SGZ, subgranular zone.

Not only are there basal differences in hippocampal neurogenesis during adolescence, but it is also a time period during which hormonal changes and alterations in lifestyle can affect neuronal proliferation and survival within the DG. Moreover, this period of the lifespan may be important in the programming of future hippocampal connectivity; thus, it is essential to better understand how potential modulators such as stress, diet and exercise could affect adolescent hippocampal neurogenesis, and subsequently have an impact upon cognitive function throughout the rest of the lifespan. This review will address the adolescent period as a critical window for the profound and lasting negative effects of stress, with a focus on how activation of the hypothalamic-pituitary-adrenal (HPA) axis and neuroinflammation may contribute to these effects. The role of puberty in producing differences between adolescent males and females in response to stress will also be discussed. Lastly, the review will consider the possible alleviation of chronic alterations to hippocampal neurogenesis and cognitive function through interventions with modifiable lifestyle factors such as exercise and diet (Figure 1).

## The impact of stress during adolescence on hippocampal neurogenesis

### Immediate effects of adolescent stress on neurogenesis

While it is well established that stress has a detrimental effect on neurogenesis in adulthood, the effect is relatively short-lived, as recovery can occur in as little as 10 days.^68^ The effects of stress during adolescence on hippocampal neurogenesis in rodent models remain relatively unexplored; however, what data there are indicate that stress during this time can produce much longer-lasting effects on neurogenesis and related cognitive function. Indeed, perhaps for pragmatic, logistical and timing reasons most experiments investigating the effects of stress in adolescence have utilised paradigms in which the final readout is during adulthood (see Table 1 for hippocampal cellular measures). The few studies in which neurogenesis measures were tested immediately while animals were still in the adolescent period have shown differing effects depending on the parameters used and examined. For example, male rats that had been exposed to social instability stress (PND30–45) had increased hippocampal cell proliferation and numbers of new neurons when examined at PND46,^69^ while female rats exposed to the same stress challenge had decreased hippocampal cell survival and no change in proliferation when examined at PND49.^70^ Male mice exposed to chronic isolation stress (5 weeks starting at PND24) exhibited a decrease in the survival of newly born cells, as well as a reduction in the proportion of newly born neurons in the cellular population when examined at PND53.^71^ Unlike the effects of social isolation in rats, no change in cell proliferation within the DG was observed.^71^ This could suggest that a longer stress challenge produces a differential response, or that the short-term effect of stress in adolescence is opposite to the effect at a later time point. In a non-human primate model, 8–10-month-old male and female marmoset monkeys showed a decrease in cell proliferation and in the number of new neurons within the DG immediately after either 1 or 3 weeks of social isolation stress.^72^ Chronic social or physical stress in male rats (4 weeks starting at PND28) did not result in changes to DG volume or neuronal density at PND56,^73^ while male rats at PND28 showed a reduced induction of long-term potentiation following elevated platform stress, suggesting impaired hippocampal function.^74^ However, DG volume, neuronal density and long-term potentiation are all indirect measures of neurogenesis, and as such may not be reflective of changes in hippocampal neurogenesis levels *per se*. Thus, from the few studies there are, it appears that the effect of stress in adolescence on neurogenesis can be either positive or negative depending on the timing of tissue collection in relation to the stress challenge and the sex of the animals tested.

### Lasting effects of adolescent stress on adult neurogenesis

The effect of stress during adolescence also appears to produce changes to hippocampal neurogenesis that last into adulthood. Male rats exposed to chronic variable physical stress for 4 weeks during adolescence (starting at PND28) showed a decrease in DG volume and an increase in DG neuronal density and DG granule cell soma size when measured 3 weeks following stressor cessation.^73^ Conversely, 4 weeks of social stress during the same period only increased DG soma size without affecting volume or neuronal density.^73^ When neurogenesis itself was measured in a similar experimental paradigm, social instability stress in male rats (PND30–45) resulted in increased numbers of immature neurons within the DG at PND74–75.^69^ In support, 4 weeks of chronic mild stress in adolescence (starting at PND30) resulted in an increase in hippocampal cell proliferation along with an increase in DG brain-derived neurotrophic factor (BDNF; a strong correlate of neurogenesis) in male rats.^76^ Interestingly, when the same stressor was applied for 4 weeks to adult male rats (starting at PND60), the opposite effect was found, with stress resulting in a decrease in cell proliferation and decreased BDNF expression.^76^ Similarly, 7 weeks of social stress starting during adolescence (PND31–33) and continuing into adulthood in male mice resulted in decreased mRNA expression of BDNF within the DG 12 months following stressor exposure.^77^ This suggests that stressor duration can have a differentially impact on neurogenesis, with the negative impact of adulthood stress potentially over-riding any pro-neurogenic effects of stress during adolescence. There also appears to be a sex difference in the impact of chronic stress on neurogenesis during adolescence, as chronic restraint stress (PND30–52) decreased hippocampal cell proliferation and survival in female rats, but increased cell survival in male rats.^75^ Thus, an overall trend for an increase in hippocampal neurogenesis in early adulthood is reported after adolescent stress exposure in males, while the opposite effect is observed in females and in males later in life. This highlights that factors such as stressor duration, the age at which it occurs, the sex of the species and the timing of tissue collection all warrant particular attention in the experimental design. As such, the factors influencing this differential response to stress-induced changes to neurogenesis across the lifespan deserves greater investigation.

### Reconciling across-studies differences in stress-induced alterations in adolescent neurogenesis

When examining the results across these studies, there are many important confounds to keep in mind, which may underlie the discrepancies observed. First, not all measurements of hippocampal function may be reflective of neurogenesis. For instance, as stated earlier, non-direct measures such as hippocampal volume and BDNF levels do not always correlate with direct cell-counting measures. An example of this is that male rats had no alterations in BDNF mRNA expression within the dorsal hippocampus, but displayed increased numbers of newly born and immature neurons within the DG 2 weeks following chronic mild stress through mid to late adolescence (PND30–58).^76^ In addition, it has recently been suggested that the marker doublecortin, which is often used as a measurement of newly born neurons, may not be a necessary mediator of neurogenesis.^78^ Thus it is important to consider the type of measurement being made when interpreting data on neurogenesis.

In addition to the types of neurogenic measurements being used, the measurement of dividing cells with the thymidine analogue 5-bromo-2'-deoxyuridine (BrdU) can vary in terms of dosage, timing of injection and timing of tissue collection post injection. For instance, lower doses of BrdU (50–100 mg kg^−1^) result in fractional labelling of mitotic cells,^79^ which could explain some of the differential results between experiments. Varied lengths of time between BrdU injection and tissue collection to assess cell proliferation have also been employed in these adolescent neurogenesis studies. While some experiments have used a 24-h interval,^71^ others used a delay of ~5 days between BrdU injection and tissue collection.^70^ In adult neurogenesis studies in which BrdU is used to label cellular proliferation, the timing between injection and cull is usually 24 h or less,^80, 81, 82, 83^ with a number of studies using a 2-h interval,^84, 85^ as this has been demonstrated to represent the timing of one labelling cycle.^86, 87^ Thus, the longer delays used in the adolescent studies discussed here may more accurately represent short-term cell survival rather than proliferation.^87^ In addition, the timing of BrdU injection in relation to the stressor onset should be considered. In some studies BrdU was administered after the stress challenge to determine the levels of cell proliferation and survival in the post-stress environment,^75, 76^ while in others the BrdU injection was done during the stressor exposure in order to examine the direct effect of stress on neurogenesis.^9, 71^ As the effects of stress can sometimes be short-lived, it is thus important to bear in mind that these differences in BrdU injection schedules could result in differential outcomes in terms of cell proliferation or survival rates. Furthermore, there is some evidence that the blood–brain barrier permeability to BrdU may vary with age. For example, it appears that the foetal brain is more susceptible to BrdU permeability,^79^ which again would make comparisons of neurogenesis difficult across the lifespan, including between adolescence and adulthood. Ideally, a dose- and time-response curve at multiple ages of BrdU labelling would be helpful in determining the optimal dosage regime of BrdU across post-natal life.

Consideration should also be given to the duration and timing of the stress challenge itself when measuring hippocampal neurogenesis responses. One of the main factors that may influence stressor effects on neurogenesis is the length of the stress challenge. Although both acute and chronic stress have a detrimental effect on hippocampal neurogenesis, in adulthood the effects of acute stress are shorter-lived.^60^ However, in adolescence shorter stress challenges may be able to produce longer effects, as evidenced by a study in which adolescent male rats were subjected to a relatively short chronic stress paradigm (15 days) altered hippocampal neurogenesis up to 30 days later.^69^

## The impact of stress during adolescence on hippocampal-dependent cognitive function

### Immediate effects of adolescent stress on cognitive function

Stress in adulthood results in deficits in cognitive behaviours such as spatial learning^88, 89^ and novel object recognition,^90, 91^ which have been shown to be dependent on hippocampal neurogenesis.^92, 93^ During adolescence, stress produces varied changes to cognition (see Table 2 for cognitive behavioural measures in rodent models) depending on the type of stressor, the cognitive test employed and the timing of the stressor. For instance, eyeblink conditioning was increased following tailshock stress during mid-adolescence (PND35–40), but not early adolescence (PND25–29) in both male and female rats.^94^ A mixed stress challenge composed of daily exposure to predator odour and elevated platform stress (PND28–30) produced increased freezing in male rats exposed to auditory fear-conditioning (PND42), but reduced freezing in a contextual fear paradigm in females at the same time point,^95^ suggesting a decrease in hippocampal-related behavioural performance. Chronic stress challenges have also been found to produce a change in cognitive function when tested in adolescence. Social isolation in male rats (30 days starting at PND25–28) resulted in impaired object recognition memory on the last day of stressor exposure.^96^ Similarly, social isolation through adolescence (5 weeks starting at PND24) resulted in impaired spatial memory in the Morris water maze (PND53–59) in male mice.^71^ Neither chronic variable physical stress nor chronic variable social stress during adolescence (4 weeks starting PND28) affected spatial memory in the Morris water maze at the end of adolescence (PND56) in male rats.^73^ Together, these studies indicate that chronic stress during adolescence may produce a negative impact on cognitive function depending on the type of stressor imposed and the cognitive test employed.

While there is evidence from human studies that indicates that stress during adolescence affects behavioural outcomes such as risk-taking,^104, 105^ there is limited evidence reporting an effect of adolescent stress on cognition in humans. However, one study has suggested that adolescents may be more vulnerable to stress-induced cognitive impairment, as adolescents exhibited an increased deficit in response inhibition under stressful conditions when compared to adults in the same paradigm.^106^ Critically, many studies of adolescent human cognition have focused on cognitive behaviours associated with cortical function such as response inhibition^106^ and cognitive control^107^ as opposed to hippocampal-dependent behaviours, and thus cannot be directly used as a measure of hippocampal function. In addition, most studies of human adolescent cognitive function have focused on the impact of early-life stress, examples of which include neglect, maltreatment and placement into foster-care, rather than the effect of stress during adolescence itself, which limits the conclusions that can be drawn. For example, adolescents with a history of caregiver deprivation who had been put into adoptive placement (starting between ages 1 and 6) had decreased cognitive control in a ‘stop-signal' task when compared to controls.^107^ While studies like this one can inform future research into the effect of stress during the adolescent period on hippocampal-based cognitive function, caution should still be taken in the interpretation of how stress during adolescence may affect cognitive function. Overall, the immediate effect of stress during adolescence on cognitive function in humans (and rodent models) remains ripe for exploration.

### Lasting effects of adolescent stress on adult cognitive function

Acute stressor exposure during adolescence can also have lasting effects on cognitive function, at least in rodent models. For example, 3 days of predator odour stress and elevated platform stress (PND28–30) increased freezing in an auditory fear-conditioning paradigm in male rats in adulthood, but produced no effects in females.^95^ This suggests that, like neurogenesis, there may be a sex difference in the cognitive response to stressor exposure during adolescence. There are also differences in the adult response depending on the timing of stressor exposure during adolescence, as variable stressor exposure in early adolescence (PND27–29) had a more pronounced negative effect in the two-way avoidance shuttle task in adulthood than did stressor exposure in mid-adolescence (PND33–35) in male rats.^101, 103^ In addition, it appears that adolescents may be more likely to develop long-term changes in cognitive function in response to stress, as adolescent male rats exposed to elevated platform stress (PND26–28) showed an increased impairment in spatial learning in the Morris water maze in adulthood when compared to rats exposed to the same stressor in adulthood.^97^ Furthermore, stressor exposure during the adolescent period may result in organisational changes that alter future responses to stress. This is evidenced by a study demonstrating that male rats exposed to elevated platform stress during adolescence, that were subsequently exposed to acute swim stress in adulthood, showed improved performance in the Morris water maze compared to either stress challenge alone.^97^ However, evidence of changes in neurogenesis in these studies has not been examined, and so to date we can only speculate on the role of acute stressor exposure during adolescence on neurogenesis and associated changes in cognition.

A larger number of studies have examined the effect of chronic stress during adolescence on adult cognitive behaviours. In contrast to acute stressor exposure, chronic mild stress for 3 weeks in mid-adolescence (starting between PND40 and 45) resulted in enhanced trace fear-conditioning in male rats, but had no effect on contextual conditioning when tested immediately following the end of stressor cessation in adulthood.^99^ Social isolation of female rats for 6 weeks (starting at PND28) impaired both object recognition and attentional set-shifting when tested at the beginning of adulthood.^100^ Similarly, chronic social instability stress during adolescence (PND30–45) resulted in impaired object location memory in both adult female^70^ and male^69^ rats. In contrast, no changes were observed when rats were tested in adolescence, suggesting that stress during adolescence may not affect behavioural outputs immediately. However, in a similar study, chronic variable physical stress, but not variable social stress, imposed for 4 weeks during adolescence (starting PND28) in male rats resulted in reduced spatial memory performance in the Morris water maze in adulthood.^73^ The reasons behind the discrepancy in these findings remains unclear, as all used social stress paradigms starting at a similar age, and utilised forms of spatial memory as the behavioural output. However, it may be that location recognition memory and spatial memory in the Morris water maze are differentially influenced upon by stress-induced deficits in hippocampal neurogenesis. The effect of chronic stress during adolescence on behaviour in aged animals has also been examined. While in one study male rats exposed to 5 weeks of chronic unpredictable stress during adolescence (from PND30 to 70) showed no changes in learning in the radial arm maze when tested 10 months later,^98^ in another, male mice exposed to 7 weeks of chronic social stress (starting approximately PND31–33) showed deficits in spatial memory in the Y-Maze and Morris water maze 12 months after the last stress exposure.^77^ Thus, the effect of stress during adolescence may not be apparent immediately, but may produce deficits throughout the lifespan, although future experiments are needed to better clarify these findings, and provide more conclusive support.

Collectively, these studies suggest that both acute and chronic stress exposure during adolescence results in long-term changes to performance in hippocampal-dependent cognitive behavioural tasks, potentially mediated through hippocampal neurogenesis. Unlike the recovery from stress observed in adulthood, which can occur in as little as 10 days,^68^ stress during adolescence can produce effects up to 12 months later. In addition, the adolescent period appears to be particularly vulnerable to certain types of stressors such as social isolation due to the evidence demonstrating that decreases in neurogenesis occurs during adolescence but not adulthood in response to this stressor.^72, 108^ However, more studies are needed to address the timing, stressor specificity and duration of these responses.

### Reconciling differences across studies into the effects of adolescent stress on cognition

As with the measurement of neurogenesis, the divergent results obtained from adolescent stress and cognition studies most likely reflect differences in experimental design across experiments. Likewise, the stressors employed in these experiments are varied in terms of both duration and severity, and as such could produce differential outcomes. For instance, as stated above, in male rats a short-term variable stressor (3 days) did not affect contextual fear-conditioning in adulthood,^95^ while a longer-term variable stressor (3 weeks) produced an increase in freezing behaviour.^99^ It is hard to pinpoint whether in this case it was the difference in the duration of stress alone that was the significant factor, as the difference in behavioural performance could also be due to the types of varied stressor used, or due to the timing of stressor exposure.

The timing of the cognitive testing following stressor exposure could have an important role in mediating the effect on behavioural outcome. It has been shown that performance in cognitive tasks changes during developmental periods.^9^ For instance, chronic variable physical stress during adolescence produced deficits in spatial memory in the Morris water maze in late adolescence but not in adulthood in male rats,^73^ whereas social instability stress impaired object location recognition only in adulthood.^69^ In addition, the same stress challenge during adolescence resulted in disparate outcomes at different adult ages. Specifically, chronic social stress during adolescence in male mice produced no change in spatial memory in the Morris water maze 6 months post stress; however, at 12 months post-stress deficits in performance were observed.^77^ Therefore, it is important to test the effects of adolescent stress on cognitive performance at a range of ages later in life in order to get a greater understanding of the lasting cognitive deficits produced in response to stressor exposure.

The tests used to measure cognitive ability in response to adolescent stress differ across reports, and could contribute to some of the diverse findings. As the adolescent period is characterised by increased impulsivity^109^ and enhanced reward sensitivity,^110^ it is important to consider potential differences in responses in cognitive tests at this time compared to adulthood. For instance, adolescent rodents are more susceptible to fear-conditioning than adults,^109^ and both adolescent rodents and humans demonstrate impaired fear extinction,^111^ which could indicate that direct comparisons between adolescents and adults on fear-motivated tasks are not appropriate. It is also imperative to note that stressor exposure has been shown to impact locomotor activity,^112^ pain susceptibility^113^ and anxiety behaviours,^114^ and as such caution should be used when interpreting the results of behavioural studies for which these changes may be confounding.

The brain circuitry required to complete various cognitive tasks differs, and this may explain some of the discrepancies in the reports on cognitive function from the literature not just in studies using adolescent rodents but in adult rodent studies also. For instance, in adult rodents, spatial memory tasks such as the Morris water maze and radial arm maze, as well as tests requiring contextual memory such as contextual fear-conditioning, have been repeatedly demonstrated to require an intact hippocampus for successful completion.^115, 116, 117, 118, 119, 120, 121^ Similarly, humans with hippocampal damage show impairments in in a virtual Morris water maze task.^122^ In tests such as novel object recognition^69, 77, 96, 100^ and cued fear-conditioning,^95, 99^ hippocampal lesion studies in adult rodents have shown mixed results, with some indicating direct hippocampal involvement,^93, 120^ and others indicating that regions such as the peri-postrhinal cortex (novel object)^118, 123^ and amygdala (cued fear-conditioning)^121^ may have a role in correct completion of the task. In the studies examining the effect of adolescent stress on cognition, the majority of those indicating a stress-induced deficit in performance utilised hippocampal-dependent spatial navigation tasks like the Morris water maze,^71, 73, 77, 97^ spatial location recognition^69, 70^ or Y-maze.^77^ Following adolescent stress animals showed either no change^69, 77^ or a decrease^96, 100^ in performance in novel object recognition, a task that may be less reliant on the hippocampus. However, other tests that are believed to be dependent on hippocampal function such as context based fear-conditioning,^95^ and radial arm maze^98^ do not result in clear cut deficits following stressor exposure during adolescence.

One explanation for this discrepancy in performance in hippocampal-dependent tasks in response to adolescent stress is that even though these tasks require hippocampal-dependent types of memory, they may not be definitively associated with changes in hippocampal neurogenesis. Tasks that require spatial memory, contextual memory and pattern separation have been shown to be dependent on hippocampal neurogenesis, at least in adult rodents.^124, 125, 126^ For example, both drug- and ablation-induced reductions in adult hippocampal neurogenesis have been shown to impair performance in trace fear-conditioning,^127^ contextual fear-conditioning,^119^ spatial memory in both the Morris water maze^37, 40^ and Barnes maze,^128^ spatial pattern separation using a radial arm maze,^36^ place learning using the Morris water maze^129^ and long-term memory in a non-matching to sample task.^130^ Lentiviral-mediated inhibition of the WNT signalling pathways (which are necessary for neurogenesis) within the DG of rats resulted in impaired spatial memory and novel object recognition,^131^ and deficits in a modified spontaneous location recognition pattern separation task.^132^ However, it has also been demonstrated that drug-induced reduction in hippocampal neurogenesis did not affect spatial learning in the Morris water maze, elevated plus maze or contextual fear-conditioning.^127^ These discrepant findings may reflect recent research, which has indicated that hippocampal neurogenesis may be involved in the forgetting of old information rather than the addition of new memories.^133^ In support, stress in early life can decrease extinction in fear-conditioning,^95^ indicative of a decline in the ability to forget a previously learned connection. Thus, the involvement of neurogenesis in cognitive tasks may be reflective of which component of memory or learning is being tested, and the type of test being used. Because of the uncertainty still surrounding the association between hippocampal neurogenesis and different types of cognitive tasks, as well as the potential for this association to change throughout the lifespan, the best course of action to assess the effect of stress on adolescent neurogenesis and cognitive function would be to correlate cognitive performance in multiple tasks carried out at various times throughout the adolescent period with measures of hippocampal neurogenesis.

## Factors that may contribute to stress-induced alternations in hippocampal neurogenesis and associated cognition during adolescence

### HPA axis response to stress during adolescence

In response to stressor exposure, the HPA axis is activated, resulting in the release of adrenocorticotropic hormone (ACTH) from the anterior pituitary gland, which in turn stimulates the release of corticosterone (CORT) from the adrenal cortex into the bloodstream. In addition, ACTH and CORT production is also stimulated by increased central release of pro-inflammatory cytokines in response to stress, especially interleukin (IL)-1β.^134^ CORT in turn signals through glucocorticoid receptors (GR) and mineralocorticoid receptors (MR) within the hippocampus to activate a negative feedback loop and clamp the HPA response.^135^ Chronically elevated CORT has long been shown to suppress neurogenesis in the adult rat DG,^136^ although both acute and chronic stress-induced increases in CORT levels by stress have also been associated with enhanced cell proliferation and survival, respectively, in male mice.^137, 138^ Moreover, the rate of hippocampal neurogenesis in the adult male rat is dependent upon circulating levels of CORT,^139^ suggesting a complex interplay between the type and duration of stressor exposure that induces CORT, as well as the critical period during the lifespan at which it occurs.^61^

Evidence suggests that during the adolescent period hippocampal negative feedback onto the HPA axis may be diminished. The HPA axis showed a longer latency to recover to baseline levels of circulating ACTH and CORT in response to acute restraint stress in adolescent (PND28) compared to adult male rats.^140^ Similarly, at PND25 male rats had a prolonged ACTH and CORT response to an ether exposure stress challenge, along with a slower rise to peak CORT concentrations compared to adult rats.^141^ Further evidence of this age difference in HPA activation comes from a study showing that adolescent male rats (PND28) did not habituate to 7 days of restraint stress, as indicated by an increased circulating CORT response, whereas adult male rats did habituate to this stress challenge.^142^ In adolescent male rats (PND31–33) exposed to the bacterial mimetic lipopolysaccharide, a blunted circulating CORT response was observed after 3 h.^143^ However, this may be reflective of a slower rise to peak concentrations of CORT similar to that which has been reported following ether stress.^141^ Interestingly, human male and female adolescents (age 13–17) also show increased HPA activation (as indicated by an increased cortisol response) in response to performance stressor exposure compared to male and female children (age 7–12) and adults.^144, 145, 146^

One of the mechanisms through which a differential HPA axis response is observed during adolescence may be a change in the corticosteroid receptor balance during this developmental period. For example, adolescent male rats exposed to variable physical stress or social stress throughout adolescence (4 weeks starting at PND28) showed reductions in GR mRNA expression within the DG.^73^ The effect of physical stress appeared more long-lasting however, as the reduction in GR mRNA within the DG was still evident in adulthood, 3 weeks after stressor cessation.^73^ As the beneficial effects of CORT on neurogenesis and cognition are believed to occur with low occupation of GR receptors and high occupation of MR,^147, 148^ alterations in the MR to GR ratio across adolescence may regulate the potential effects of CORT at this time. Indeed, both male and female rats show an increase in MR and GR gene expression within the hippocampus during the adolescent period; mRNA levels of both receptors increase between PND15 and PND35 and again from PND60.^149^ In marmoset monkeys the trend is reversed, with mRNA levels of MR and GR declining from infancy through adolescence to adulthood.^150^ While there are reported increases in GR mRNA expression in the prefrontal cortex in humans during adolescence (age 14–18), no changes have been reported in the hippocampus.^151^ Thus, although GR expression is also decreased by stress in adulthood,^152^ the altered basal MR/GR expression during adolescence could result in differential changes to the glucocorticoid system within the hippocampus following stress at this time. In addition, the alterations in MR and GR ratios may affect the negative feedback imposed by the hippocampus on the HPA axis in response to stress. Thus, the increased HPA and CORT response to stress in adolescence may be a mechanism through which stressor exposure at this time of life may exert long-lasting effects on both hippocampal neurogenesis and cognition.

### Neuroinflammatory response to stress during adolescence

Stressor exposure also results in increased expression of pro-inflammatory cytokines such as IL-1β,^153, 154^ IL-6,^155, 156, 157^ and tumour necrosis factor α,^158, 159, 160^ which have detrimental effects on hippocampal neurogenesis.^161^ Specifically, exposure to these cytokines has been shown to decrease the proliferation,^154, 155, 160^ survival^157, 159^ and neuronal differentiation^153, 156, 158^ of new cells in the embryonic and adult hippocampus. In addition to the HPA axis regulation, CORT also signals through GR and MR in the hippocampus to negatively feedback on the inflammatory response, reducing IL-1β expression.^162, 163, 164^ Under conditions of severe and chronic stressor exposure however, not only does the HPA axis remain activated, but a neuroinflammatory milieu ensues within many brain structures including the hypothalamus and hippocampus.^165, 166, 167, 168, 169, 170, 171, 172^

There is some evidence to suggest that there may be differences in inflammatory signalling pathways responsible for regulating neurogenesis across the lifespan, especially during the adolescent period.^173^ For example, *in vitro* studies have shown that in neural progenitor cells (NPC) cultured from adolescent mouse hippocampus (PND21), administration of IL-1α promoted an increase in cell proliferation, while NPC cultured from adult murine hippocampus did not.^155^ However, cell proliferation was inhibited by IL-6 administration in NPCs prepared from both adolescent and adult hippocampi.^174^ Likewise, another report has indicated that, *in vivo,* the cellular response to IL-1β may change across the lifespan. Adult male mice (5 months) overexpressing the IL-1 receptor antagonist showed decreased hippocampal cell proliferation compared to wild types, yet this difference disappeared with ageing (22 months).^175^ However, it remains to be determined whether there are differential cytokine signalling pathways that have an impact upon neurogenesis during adolescence compared to adulthood, and indeed whether there are sex differences in the response to inflammatory-induced changes in neurogenesis and associated cognition.

### Sex differences in the response to stress during adolescence

Sex is an important contributor to differences in basal levels of adult hippocampal neurogenesis,^85, 176, 177, 178^ for example, at PND35 male rats exhibit higher levels of cellular proliferation in the DG than females,^179^ while adult female rats have higher levels of proliferation than males.^176^ Moreover, as there are bi-directional interactions between the HPA and HPG axes starting during puberty, which allow for sex-dependent stress responses,^180, 181, 182, 183^ it is important to consider sex differences in the response to stress during adolescence in rodents, and that these responses may differ from those seen in adulthood. For example, in two reports from the same group, similar experimental design and tissue collection points were employed for both male and female rats. In one experiment, adolescent female rats showed decreased cell proliferation in the DG following social instability stress (PND30–45),^70^ while in another, male rats showed an increase in cell proliferation and numbers of new neurons within the DG at the same time point.^69^ This also corresponds with the finding that following chronic restraint stress in adolescents (PND30–52), female rats had decreased cell survival, whereas male rats showed increased cellular survival within the DG.^75^ Interestingly, these changes are opposite to those observed in adulthood, as adult male rats show a decrease in neuronal proliferation following acute stress^184^ as well as a decrease in survival of new neurons after chronic stress.^185^ In adult female rats, however, an increase in the survival of new neurons, and no change in proliferation, was observed following chronic stress.^185^ These results suggest that adolescent females may have an inherent vulnerability to chronic stress compared to adolescent males, while adult females appear to be resistant to the detrimental effects of chronic stress on hippocampal neurogenesis. The differences between males and females in the neurogenic response to stress may be due to alterations in circulating hormone levels,^39, 186^ as adult female rats show an increase in the number of proliferating cells during the pro-oestrus phase of the oestrus cycle,^176^ a time characterised by high oestrogen levels. In support, it has been reported that gonadectomised adult female rats treated with oestrogen show increased cell proliferation,^85, 177^ but decreased neuronal survival within the DG,^177^ while castrated adult male rats show decreased survival of newly born cells, an effect that is reversed with testosterone replacement as well as with the testosterone metabolite dihydrotestosterone.^178^ The mechanism underlying the effects of steroid hormones on neurogenesis is due to the prevalence of oestrogen,^187^ androgen^188^ and progesterone^189^ receptors on neural stem cells in the hippocampus.^39, 186^ Steroid hormones and their interaction with cognate receptors on neural stem cells are critical for the organisational programming of the brain during the pubertal period of adolescence.^38, 190, 191^ Thus, the impact of stress on the HPA axis during adolescence, particularly during the pubertal period in females, may produce lasting changes to the HPG system and consequently organisational changes to hippocampal structures.

Although there is limited evidence to show that HPG axis hormones can cause differences in cognitive function in adolescent rodents,^192, 193, 194, 195, 196^ the literature includes reports from studies using adult rodents. For example, gonadectomy in adult male rats resulted in impaired performance in an inhibitory-avoidance task, suggesting a role for testosterone in increasing hippocampal-dependent cognition.^197^ In female rodents, treatment with either progesterone or oestrogen following ovariectomy resulted in increased performance on a novel object task.^198^ Moreover, it has been demonstrated that hippocampal-dependent cognition, especially spatial learning and memory, can vary across the oestrus cycle, with performance waning during the oestrus phase in mice following the peak of oestrogen levels.^199^ Similarly, in human females, spatial cognitive ability was decreased during the follicular and luteal phases when oestrogen levels were low.^200, 201^ McCormick *et al.*^69, 70^ have attempted to explain the potential differences in neurogenesis-associated cognitive function between adolescent males and females in response to social instability stress. In their experiments, they showed that mid-adolescent male rats (PND46) had an increase in the number of new neurons within the DG in response to social instability stress,^69^ whereas females (PND49) had a reduction in the number of new cells at a similar time point,^70^ and neither sex had a change in cell proliferation.^69, 70^ However, both male and female adolescent rats showed no change in spatial location memory when tested during the adolescent period, but a decrease in memory performance when tested in adulthood.^69, 70^ While there were some discrepancies in the measures of neurogenesis used and the timing of tissue collection between these two studies, overall they suggest that, while males and females may have differences in the immediate response to stress, the lasting negative impact on cognitive function is not discrepant across sexes. In support, both male and female adolescent rats show an increase in trace eyeblink conditioning following footshock,^94^ indicating that the cognitive response to stress during adolescence may not differ between males and females. However, there is some evidence that suggests that females may be the more susceptible sex to the negative effects of stress on hippocampal-dependant cognition. Adolescent male rats showed an increase in auditory fear-conditioning following chronic variable stress, while females were impaired in a contextual fear-conditioning task,^95^ a task that is more reliant on hippocampal neurogenesis. Thus, it will be important in future studies of adolescent hippocampal neurogenesis and cognitive function to note the pubertal state of the animals and the phase of the oestrus cycle not only at the time of cognitive testing, but also at the time of stress challenge and tissue collection.

## Potential interventions during adolescence that could ameliorate stress-induced impairments in hippocampal neurogenesis and cognition

Epidemiological studies have revealed that human adolescence is often associated with altered meal intake patterns and changes in physical activity levels.^202^ Studies have indicated that less than 50% of teens receive the daily-recommended intake of fruit, vegetable, dairy or protein, while intake of fats and sugars during adolescence is well above the recommended levels.^203, 204^ In addition, adolescents are more likely to engage in unhealthy behaviours such as dieting and weight-control behaviours,^205^ as well as increased alcohol and drug use.^206, 207, 208^ Along with the reduction in healthy eating behaviours during this period, adolescence has also been shown to be a time of reduced physical activity,^209^ especially moderate-to-vigorous activity.^210^ This is in parallel to an increase in sedentary behaviours, such as TV viewing, video game and computer activity, and increased homework and reading time.^211^ In addition, unhealthy diet, increased alcohol intake and decreased exercise levels have been associated with decreased hippocampal neurogenesis and cognitive performance in rodent models;^66, 212, 213, 214, 215^ thus, it is possible that the lifestyle changes often associated with adolescence may have a negative impact on hippocampal function. Adolescence is also a critical time for the programming of patterns of behaviour, such as diet intake and exercise, which will carry over to adulthood,^3^ and as such dietary and exercise interventions during this period have the potential to alter hippocampal function and cognition throughout the lifespan, not just in adolescence itself. It should be noted, however, that due to the current scarcity of evidence from human cohorts, data from rodent studies should be viewed as informative rather than strictly translational.

### Exercise

In adult rodents, aerobic exercise increases synaptic strength and plasticity, learning and growth factors within the hippocampus,^67, 216^ and also increases neurogenesis within the DG.^66, 80, 217^ It has previously been shown that 1 week of forced low-intensity running during adolescence in male rats (starting approximately PND35) resulted in increased survival of BrdU-labelled cells, an increased percentage of new cells differentiating into neurons and increased hippocampal BDNF mRNA expression^218^ compared to sedentary rats. Similarly, male and female rats trained on a rotarod from PND35 to 38 showed increased cell survival at PND49 as measured by the number of BrdU-labelled cells.^219^ In support, 8 weeks of forced running in male rats (starting at PND22) resulted in increased performance in the Morris water maze and increased neuronal numbers measured with cresyl-violet staining throughout the hippocampus and DG, which may suggest increased neurogenesis.^220^ Male rats subjected to forced running for 5 weeks (from PND21 to PND60) also showed increased mossy fibre density and BDNF protein levels within the hippocampus, along with increased spatial memory in the Morris water maze.^221^ Voluntary exercise can also have an impact upon neurogenesis; 13 days of voluntary running during adolescence (PND30–42) by male rats produced increases in both cell proliferation and survival within the DG.^222^ Moreover, 4 weeks of voluntary access to a running wheel during adolescence (starting PND31) increased performance in the novel object recognition task in male rats, an effect that was still observed 4 weeks after the exposure to exercise ended.^223^ In the same paradigm during adulthood, the immediate positive impact of voluntary running on object recognition in male rats had diminished within 2 weeks of post-running wheel exposure.^223^ In humans, physical fitness during adolescence (~14 years of age) is positively correlated to cognitive performance, while an acute bout of exercise (20  min of graded cycling) produced no changes.^224^ It should be noted that both acute and chronic exercise have been linked to increased executive function,^225, 226^ although this most likely indicates prefrontal cortex rather than hippocampal changes. However, a positive correlation has also been observed between aerobic activity and both hippocampal size and cognitive performance on a virtual Morris water maze, indicating that the link between exercise and hippocampal-dependent tasks is also evident in human adolescents (~16 years of age).^227^

There is some evidence that exercise during adolescence ameliorates the deleterious effects of early-life stress on cognitive behaviours and neurogenesis. Voluntary running in adolescent male mice (PND21–52) reversed the negative cognitive effects of prenatal stress in the Morris water maze, and also increased granular cell dendritic length and intersections within the DG, which suggests a recovery of cell maturation.^228^ Environmental enrichment studies in adult rodents (in which they are housed in larger cages, in larger groups, with access to toys, tunnels, besting material and with opportunity to exercise on running wheels^229^ have demonstrated that exercise is the critical element of environmental enrichment-induced enhancement of hippocampal neurogenesis and cognitive performance.^230, 231^ While the effect of exposure to an enriched environment during adolescence on hippocampal neurogenesis has not been yet reported in the literature, it has been shown that environmental enrichment, including free access to a running wheel (starting at PND22), attenuated the increase in body weight gain and deficit in social behaviours observed following prenatal stress in male rats.^232^ In addition, enrichment with access to running wheels (starting PND30) immediately following stressor exposure during adolescence (PND27–29) attenuated the stress-induced anxiety-like behaviours, altered HPA axis tone and impaired avoidance learning in male rats.^102^ Although the beneficial effects of environmental enrichment in adulthood on hippocampal neurogenesis has been shown to be largely due to increased physical activity,^230, 231^ it remains to be determined whether the same holds true during the adolescent period. Taken together, despite the heterogeneity of types of exercise employed, these studies indicate a positive effect of exercise, especially aerobic exercise, on hippocampal neurogenesis and cognitive function in adolescence. This suggests that exercise could act to reverse the negative impact of adolescent stress. However, there is a lack of direct evidence for the benefit of exercise in modulating stress-induced effects in adolescence compared to adulthood, and as such future studies should investigate whether there is an age-dependent response, and through which mechanisms these changes are occurring.

### Mechanisms underlying exercise-induced changes to hippocampal neurogenesis and cognition

Many mechanisms underlying the effects of exercise on hippocampal function, including its potent effect on hippocampal neurogenesis in adulthood, have been explored using rodent models; however, the mechanism in adolescence may be different. For example, it has been found that, in male rats, forced treadmill running during adolescence (PND21–60) decreases cannabinoid receptor expression throughout the brain, including within the hippocampus.^233^ This is opposite to the effect in adulthood, where voluntary running increased cannabinoid receptor expression within the hippocampus of male rats.^234^ As the endocannabinoid system has been demonstrated to modulate hippocampal neurogenesis,^235^ especially the proliferation and migration of neurons,^235, 236, 237^ it may be that alterations to this system during a period of brain remodelling such as adolescence can produce long-lasting alterations to hippocampal structure and function that are different than those seen in adulthood. Moreover, the endocannabinoid system has been linked to HPA axis function, in that it helps to maintain homeostasis and habituation of the HPA axis.^238^ Thus, the endocannabinoid system may be one mechanism through which exercise can alter the effects of stress on neurogenesis, especially during adolescence.

The BDNF system has also been shown to be a major mechanism underlying the positive effects of exercise on neurogenesis.^239^ Moreover, BDNF has been shown to be protective against the negative effects of stress on neurogenesis and cognition.^240^ Forced running during the late adolescent period (PND 41–50) in male rats produces heightened levels of BDNF in the hippocampus in response to high-intensity exercise.^241^ Because expression of the BDNF receptor TrkB decreases during the course of the lifespan,^242^ it could be that increased levels of this growth factor could have a greater effect during adolescence than in adulthood. Adolescence may thus represent a time during which exercise and the subsequent elevation of BDNF expression within the hippocampus could produce a more potent and longer-lasting attenuation of stress-induced decreases in neurogenesis and cognition compared to adulthood. In addition, there could be sex differences in the effect of exercise on BDNF levels, as the BDNF gene contains an oestrogen-response element^243^ and increased concentrations of oestrogen result in heightened levels of BDNF expression.^244, 245^ In support of this, a reduction in the exercise-induced increase in hippocampal BDNF expression in ovariectomised adult rats has previously been reported.^246^ As such, the changes in circulating oestrogens after puberty could contribute to a differential effect of exercise on BDNF levels, and consequently on hippocampal neurogenesis and associated cognition in adolescent males and females. However, more experiments are necessary to better elucidate the mechanisms underlying the amelioration of stress-induced effects by exercise, to further examine the differences between these mechanisms in adolescence in comparison to adulthood, and to determine whether there are sex differences in their actions.

Voluntary running has also been shown to alter the microbiota of adult male rats,^247, 248^ increasing the diversity of bacteria present. Recently, studies have begun to emerge that suggest a role for the microbiota in regulating both hippocampal neurogenesis^249^ and cognitive function.^250^ Interestingly, changes to the microbiota in adult female mice resulted in impaired hippocampal neurogenesis and decreased cognitive performance in a novel object recognition task, an effect that was rescued by 10 days of voluntary running.^251^ Thus, it appears that there is a relationship between exercise, the microbiota and neurogenesis, and as such microbiota changes may be one mechanism through which exercise can exert effects on hippocampal function and cognition in adolescence. In addition, it has also been demonstrated that adolescent humans have a differing microbiota diversity, with higher abundance of some genera, such as *Bifidobacterium* and *Clostridium* compared to adults.^252^ While in rodents early-life maternal separation stress results in changes to both the microbiota^113^ and deficits in cognitive performance,^253^ the effects during adolescence remain unclear. Interestingly, alterations of the microbiota during adolescence modifies hippocampal BDNF levels and decreases cognitive performance in the novel object recognition task in male mice.^254^ However, the role of the microbiota in mediating the effects of adolescent stress, as well as the potential role of exercise as a positive modulator during this critical window, is an exciting new mechanism to explore.

### Dietary changes

Diet is one of the most easily modifiable lifestyle factors that is known to alter cognitive and behavioural performance, as well as neurogenesis.^63, 64, 65^ A growing body of evidence now points to the importance of adolescent diet for general well-being, including brain health.^255, 256^ Moreover, changes in the human diet over the past 50 years are potentially putting the adolescent brain in a more vulnerable state. ‘Western' diets high in processed foods, fats and sugars result in many problems including obesity,^257, 258^ diabetes^259^ and cognitive and emotional disorders.^260, 261^ On the other hand, healthy diets, such as a Mediterranean diet high in vegetables, fish, fruits and nuts provide many macronutrients such as omega-3 fatty acids, omega-6 polyunsaturated fatty acids and flavonoids,^262^ which promote both brain^263, 264^ and body health.^265^ Thus, the evidence to date suggests that not only is the nutritional status important for adolescent brain health, but so is the overall diet composition including macronutrients, fatty acids and vitamin levels.

To date, most studies examining the effect of diet during adolescence on cognition and neurogenesis have focused on the detrimental effect of high-fat diets (HFD) or high-sugar diets, rather than any beneficial effect of lower calorie or nutrient-supplemented diet. For example, in rodent models, male mice fed a HFD for 11 weeks throughout adolescence (starting at PND21) exhibited decreased cognitive performance in the radial arm maze, and decreased numbers of new neurons in the DG, while neither of these changes were observed when the diet was administered during adulthood.^213^ Similarly, a HFD fed to male rats during adolescence (for 11 weeks starting at PND21) decreased long-term memory performance in the Morris water maze, while a HFD during adulthood did not result in any of these changes.^266^ In addition, human adolescents consuming ‘Western' diets have decreased performance in visual-spatial learning and memory tasks, both of which are associated with hippocampal function.^267^ In support of the link between nutritional state and hippocampal function, consumption of diets lacking in Vitamin A and E resulted in decreased proliferation of new neurons in the DG^268, 269^ and reductions in spatial learning and memory^268, 270^ in adult male rats.

Some studies have examined the positive impact of nutrition on hippocampal neurogenesis and cognition in adulthood.^63^,^64^ Low-calorie diets have been shown to increase memory performance in aged human subjects.^271^ Findings from studies using adult male and female rodents suggest that low-calorie diets^272, 273, 274, 275, 276^ or diets with increased flavonoids,^277, 278, 279^ omega-3 fatty acids,^280, 281, 282^ polyphenols,^283^ magnesium^284^ or zinc^285^ can increase proliferation and survival of newly born neurons in the DG,^274, 275, 276, 278, 279, 281, 282, 283, 285^ as well as performance on hippocampal-related learning and memory tasks.^272, 273, 277, 280, 284^ There is also some evidence to suggest that nutritionally beneficial diets can protect against stress-induced impairment in hippocampal neurogenesis as well as cognitive deficits. For example, treatment of neuronal cultures with an omega-3 fatty acid reversed the CORT-induced prevention of neuronal differentiation.^286^ In addition, flavonoid-rich diets administered to rats (sex unspecified) that were subjected to chronic unpredictable stress reversed the stress-induced decrease in proliferating new neurons within the DG.^287^ Similarly, male and female mice subjected to 10 days of calorie restriction show attenuation of chronic social stress-induced depressive-like behaviours.^288^ Together, these results indicate that healthier diet interventions may have an important role in regulating hippocampal function. However, as with exercise, whether there is a differential effect of diet on stress-induced changes in hippocampal neurogenesis and cognition during adolescence compared to adulthood remains to be seen.

### Mechanisms underlying diet-induced changes to hippocampal neurogenesis

BDNF also appears to be a critical mediator for the beneficial effects of diet on neurogenesis and cognitive function. For example, diets high in zinc have been shown to result in increased BDNF mRNA and protein expression within the hippocampus of adult male rats.^289^ In addition, the dietary restriction-related increase in the survival of newly born neurons within the DG in wild-type mice was attenuated in BDNF^+/-^ adult male mice, suggesting a role for BDNF in mediating this effect.^274^ While there is no evidence in the literature to date on the potential role of BDNF in dietary-induced protection against changes to neurogenesis and cognition evoked by stress, there is some evidence to suggest that dietary changes may protect against brain damage through BDNF pathways. For instance, in adult male rats a diet high in omega-3 fatty acids or curcumin were protective against an injury-induced deficit in both hippocampal BDNF expression and performance in the Morris water maze.^290, 291^ While in these studies a physical brain injury stressor was used, the findings hint at a potential protective role of dietary-induced BDNF increases in response to other stress challenges, at least in adulthood. As BDNF-induced pro-neurogenic and pro-cognitive effects may be more pronounced during adolescence than adulthood,^242^ this suggests that a healthy diet, like exercise, may be especially beneficial for hippocampal development and function during this period of the lifespan than in later years. It may thus be the case that changes to diet during adolescence may produce longer-lasting effects than when a nutritional diet commences during adulthood.

It should also be noted that diet is the leading factor that induces changes in gut microbiota composition,^292, 293, 294, 295^ and the negative effect of stress on the microbiome in early life^113, 296^ and adulthood^297^ can be reversed by dietary changes. As with exercise, the effect of stress and diet on the microbiome has not yet been studied in adolescence, although it is known that there are changes to the gut microbiota structure and balance during the adolescent period.^298^ However, the influence of many of the lifestyle changes that begin during the adolescent period including stress, diet, alcohol and drug consumption has an impact on the microbiome, and thus it could be an important mechanism underlying hippocampal neurogenesis regulation and associated cognitive function during this time of the lifespan.^299, 300^

These findings suggest that, while adolescence may represent a period during which stress may have a more profound and long-lasting effect on hippocampal neurogenesis and cognitive function, it could also be a time during which positive changes such as healthier diet and increased exercise could not only combat these detrimental effects, but produce positive effects in their own right. Thus, future research into the potential synergistic effects of diet, exercise and stress are needed, and will help to determine which interventions during adolescence produce significant effects on cognition in later life.

## Conclusions

Adolescence represents a unique period of the lifespan in which there is considerable re-organisation and growth of many brain structures, including the hippocampus. During this time there is increased hippocampal neurogenesis, and an altered HPA and inflammatory response to stress. As such, stress during this time of life may produce differential and long-lasting detrimental changes in the hippocampus that could last throughout adulthood. Specifically, stress-induced changes in hippocampal neurogenesis could lead to long-lasting deficits in cognitive function, especially in spatial and context related tasks. The pubertal onset of circulating HPG axis hormones leads to the onset of sex differences in the response to stress-induced impairments in hippocampal neurogenesis and cognition, with the scant data to date indicating that females are more vulnerable to stress. With growing evidence that the hippocampus is particularly responsive to environmental and lifestyle influences during adolescence, we hypothesise that intervention with positive factors such as a healthy diet and exercise may prevent the negative impact of stress during adolescence on hippocampal neurogenesis and cognitive function not only during the adolescent period itself, but throughout the rest of the lifespan. Moreover, maintaining a healthy diet and regular exercise during adolescence may help to programme these positive behaviours for the rest of the individual's lifespan.

However, much work is still needed to fully elucidate both the immediate and lasting impact of stress during adolescence on hippocampal neurogenesis and associated cognitive function, as well as the potential role for diet and exercise in attenuating these effects. Future research should address the adolescent period as a key window during which programming of hippocampal neurogenesis occurs, and focus on better understanding the mechanisms, such as the endocannabinoid system, BDNF and the gut microbiota, underlying stress-induced changes at this time. In addition, the effect of the onset of sex differences in the neurogenic and cognitive response to stress challenges should be examined, as well as the role of diet and exercise in modulating these changes. To better capture the effects of stress, diet and exercise, multiple measures of hippocampal neurogenesis and hippocampal-dependent cognitive behaviours should be investigated. Finally, it is important to clarify the translational impact of these findings through further study of hippocampal-dependent cognitive processes in the human adolescent in response to these modifiable lifestyle factors.

## Figures and Tables

**Table 1:** Summary of the effects of adolescent stress on hippocampal neurogenesis and neuroplasticity measures in rodent models

*Stressor*	*Species*	*Sex*	*Age (PND) at*			*Effect on hippocampal neurogenesis and neural plasticity*	*References*
			*Stress*	*BrdU (i.p.)*	*Tissue collection*		
Elevated platform	Wistar rat	M	28	N/A	28	↓ LTP in CA1	Xiong *et al.*^74^
					28	↑ LTD in CA1	
Chronic restraint	SD rat	M	30–52	70	91	↔ Cell proliferation (Ki67)	Barha *et al.*^75^
					91	↑ Cell survival (BrdU)	
					91	↔ Neuronal differentiation (Brdu/NeuN); DG volume	
Chronic restraint	SD rat	F	30–52	70	91	↓ Cell proliferation (Ki67)	
					91	↓ Cell survival (BrdU)	
					91	↔ Neuronal differentiation (Brdu/NeuN); DG volume	
Chronic mild	SD rat	M	30–58	72	77	↑ Number new cells (BrdU)	Toth *et al.*^76^
					77	↔ Number of new neurons (BrdU/DCX)	
					77	↑ BDNF protein in DG	
Chronic physical	SD rat	M	28–55	N/A	56	↑ Volume of CA1	Isgor *et al.*^73^
					56	↔ Volume; neuron number; neuronal soma size in CA3, DG	
					76	↓ Volume of CA1, CA3, DG	
					76	↑ Neuronal soma size in CA1, DG	
					76	↔ Neuron number	
Chronic social	SD rat	M	28–55	N/A	56	↑ Volume of CA1	
					56	↔ Volume; neuron number; neuronal soma size in CA3, DG	
					76	↑ Neuronal soma size in CA1, DG	
					76	↔ Volume; neuron number in CA1, CA3, DG	
Chronic social	CD1 mouse	M	~32–80	N/A	~450	↓ BDNF in CA1, CA3, DG	Sterlemann *et al.*^77^
					~450	↓ DG synaptic density (synaptophysin protein)	
					~450	↓ LTP in CA1	
Social instability	LE rat	M	30–45	N/A	33	↑ Cell proliferation (Ki67)	McCormick *et al.*^69^
					46	↔ Cell proliferation (Ki67)	
					46	↑ Number of new neurons (DCX)	
					74/75	↔ Cell proliferation (Ki67)	
					74/75	↑ Number of new neurons (DCX)	
Social instability	LE rat	F	30–45	43–45	49	↔ Cell proliferation (Ki67)	McCormick *et al.*^70^
					49	↓ Number of new cells (BrdU)	
Social isolation	ICR mouse	M	24–52	23	53	↓ Cell survival (BrdU); neuronal differentiation (BrdU/NeuN)	Ibi *et al.*^71^
				52	53	↔ Cell proliferation (BrdU)	

Abbreviations: BrdU, bromodeoxyuridine; BDNF, brain-derived neurotrophic factor; CA1, Cornus ammonis region 1 of the hippocampus; CA3, Cornus ammonis region 3 of the hippocampus; DCX, doublecortin; DG, dentate gyrus; F, female; ICR, Institute for Cancer Research; i.p., intraperitoneal; LE, long-Evans; LH, Lister Hooded; LTD, long-term depression; LTP, long-term potentiation; M, male; N/A, not applicable; NeuN, neuronal nuclei; PND, post-natal day; SD, Sprague–Dawley.

**Table 2:** Summary of the effects of adolescent stress on hippocampal-related cognitive behaviours in rodent models

*Stressor*	*Species*	*Sex*	*Age (PND) at*		*Cognitive test*	*Cognitive effect*	*Reference*
			*Stress*	*Testing*			
Elevated platform	Wistar rat	M	26–28	60	MWM	↓ Learning	Avital *et al.*^97^
			28	90	MWM	↑ Reversal	
Tailshock	SD rat	M	~27	~27–28	TEC	↔ Responses	Hodes *et al.*^94^
			~37	~37–38	TEC	↑ Responses	
Tailshock	SD rat	F	~27	~27–28	TEC	↔ Responses	
			~37	~37–38	TEC	↑ Responses	
Chronic mild	SD rat	M	30–70	~385	RAM	↔ Latency	Chaby *et al.*^98^
Chronic mild	SD rat	M	~43–63	~64–67	TFC	↑ Freezing	Reich *et al.*^99^
				~64–67	DFC	↔ Freezing	
Chronic physical	SD rat	M	28–55	56	MWM	↔ Learning	Isgor *et al.*^73^
			28–55	76	MWM	↓ Learning	
Chronic social	SD rat	M	28–55	56	MWM	↔ Learning	
			28–55	76	MWM	↔ Learning	
Chronic social	CD1 mouse	M	~32–80	~270	MWM	↔ Learning	Sterlemann *et al.*^77^
				~440	MWM	↓ Learning	
				~440	Y-Maze	↓ Learning	
				~440	NOR	↔ Recognition	
				~440	SDL	↔ Recognition	
Social instability	LE rat	F	30–45	46–48	SLR	↔ Memory	McCormick *et al.*^70^
				70–72	SLR	↓ Memory	
Social instability	LE rat	M	30–45	46–49	SLR	↔ Memory	McCormick *et al.*^69^
				46–49	NOR	↔ Recognition	
				70–73	SLR	↓ Memory	
				70–73	NOR	↔ Recognition	
Social isolation	ICR mouse	M	24–59	53–59	MWM	↓ Learning	Ibi *et al.*^71^
Social isolation	LH rat	M	~28–58	~58	NOR	↓ Recognition	Bianchi *et al.*^96^
Social isolation	LH rat	F	28–70	~70	NOR	↓ Recognition	McLean *et al.*^100^
				~70	AS	↓ Learning	
Variable	SD rat	M	27–29	59–60	TWS	↓ Avoidance	Tsoory *et al.*^101^
			33–35	59–60	TWS	↓ Avoidance	
Variable	SD rat	M	27–29	60–61	TWS	↓ Avoidance	Ilin *et al.*^102^
Variable	SD rat	M	27–29	63	TWS	↓ Avoidance	Tsoory *et al.*^103^
Variable	WH rat	M	28–30	41–42	CFC	↔ Freezing	Toledo-Rodriguez *et al.*^95^
				41–42	AFC	↑ Freezing	
				83–87	CFC	↔ Freezing	
				83–87	AFC	↑ Freezing	
Variable	WH rat	F	28–30	41–42	CFC	↓ Freezing	
				41–42	AFC	↔ Freezing	
				83–87	CFC	↔ Freezing	
				83–87	AFC	↔ Freezing	

Abbreviations: AFC, auditory fear-conditioning; AS, attentional set-shifting; CFC, contextual fear-conditioning; DFC, delay fear-conditioning; F, female; ICR, Institute for Cancer Research; LE, long-Evans; LH, lister hooded; M, male; MWM, Morris water maze; NOR, novel object recognition; PND, post-natal day; RAM, radial arm maze; SD, Sprague–Dawley; SDL, social discrimination learning; SLR, spatial location recognition; TEC, trace eyeblink conditioning; TFC, trace fear-conditioning; TWS, two-way shuttlebox; WH, Wistar Han.
